# Homocrystallization and Stereocomplex Crystallization Behaviors of As-Spun and Hot-Drawn Poly(l-lactide)/Poly(d-lactide) Blended Fibers During Heating

**DOI:** 10.3390/polym11091502

**Published:** 2019-09-14

**Authors:** Tien-Wei Shyr, Huan-Chieh Ko, Hsin-Lung Chen

**Affiliations:** 1 Department of Fiber and Composite Materials, Feng Chia University, Taichung 40724, Taiwan; hcko@mail.fcu.edu.tw; 2 Department of Chemical Engineering, National Tsing Hua University, Hsin-Chu 30013, Taiwan; hlchenpoly@gmail.com

**Keywords:** PLLA/PDLA blended fiber, homocrystallization, stereocomplex crystallization, MDSC, HSPM, real-time WAXD

## Abstract

A series of poly(l-lactide)/poly(d-lactide) blended chips (LD_C_), as-spun LD fibers (LD_A_) and hot-drawn LD fibers (LD_H_) were prepared for investigating the homocrystallization and stereocomplex crystallization behaviors of LD_A_ and LD_H_ fibers during heating. Modulated differential scanning calorimetry (MDSC), hot stage polarized microscopy (HSPM), and real-time wide-angle X-ray diffraction (WAXD) were used for studying the crystallization and melting behaviors, fiber morphology, and crystalline structure evolution of the LD_A_ and LD_H_ fibers’ homocrystals and stereocomplex crystals during heating. The molecular chain orientations of the LD_A_ and LD_H_ fibers were obtained through spinning and improved through the hot drawing processes. When the molecular chain was oriented on the fiber axis, the homocrystals and stereocomplex crystals of the fibers began to form in turn as the heating temperature exceeded the glass transition temperature of the fiber. The side-by-side packing of the molecular chains was promoted by mixing the molecular chains with the extrusion screw during the spinning process, facilitating stereocomplex crystallization. When the LD_A_ fiber was heated above the glass transition temperature of the fiber, movement of the fiber molecular chain—including molecular chain orientation and relaxation, as well as crystallization, melting, and recrystallization of homocrystals and stereocomplex crystals—were investigated through HSPM. MDSC and real-time WAXD were used to observe the molecular chains of the melted poly(l-lactide) and poly(d-lactide) homocrystals of the fibers rearranging and transiting to form stereocomplex crystals during heating.

## 1. Introduction

Polylactide (PLA) is an aliphatic polyester with excellent biocompatibility and biodegradability. Monomer, lactic acid exists in two enantiomeric forms: l-lactic acid and d-lactic acid. Blending poly(l-lactide) (PLLA) with poly(d-lactide) (PDLA) results in l- and d- molecular chains packed side by side to form stereocomplex crystals or consisting solely of l- or d- molecular chains to form homocrystals [1,2]. The structure of a stereocomplex crystal is different from that of a homocrystal, and the melting temperature of a stereocomplex crystal is higher than that of a PLLA homocrystal. Tsuji et al. [3,4] reported that the maximum quantity of stereocomplex crystals is formed at a 1:1 weight ratio of PLLA:PDLA. The stereocomplex crystals present in the blend served as a nucleating agent, significantly increasing the number of PLLA spherulites per unit of area or volume and enabling the acceleration of the overall PLLA crystallization. Shyr et al. [5] also reported that a single endothermic peak related to stereocomplex crystals could be obtained after remelting and recrystallization of PLLA:PDLA in a 1:1 weight ratio. The regime II→III transition of the stereocomplex crystals in the poly(l-lactide)/poly(d-lactide) (LD) blends was determined to be 165 °C. The growth of concentric spherulites composed of homocrystals and stereocomplex crystals revealed that stereocomplex crystals formed in the first-stage growth acted as a nucleating agent.

The homocrystal (α-form) to stereocomplex crystal transition was identified as a melting and recrystallization process. Fujita et al. [6] reported that the homocrystal of a binary PLLA/PDLA mixture exhibits the reorganization to stereocomplex crystals caused by both chain diffusions in the solid state and partial melting and recrystallization during the annealing process. In orientated PLLA/PDLA samples, Xiong et al. [7] argued that the stereocomplex crystal comes from amorphous regions and molten α crystal. Na et al. [8] reported that the crystallization of stereocomplex crystals from molten α′ crystals is faster than that from α crystals and explained that α′ crystals have smaller aggregations and higher stereocomplex crystal nucleation density. Yin et al. [9] proposed that stereocomplex crystals are formed mainly through recrystallization of molten homocrystals. The chain diffusion between PLLA-rich domains and PDLA-rich domains might play a major role. The existing stereocomplex crystal acts as a diffusion barrier hindering further chain diffusion. Even in the PLLA/PDLA blended fibers, the annealing forms a fiber consisting mainly of highly oriented stereocomplex crystals. Takasak et al. [10] obtained highly oriented and highly crystallized fibers containing homocrystals and stereocomplex crystals through high-speed melt spinning of racemate PLA. Drawing and annealing of the as-spun fibers revealed that the starting structure, with a certain quantity of stereocomplex crystals, could be utilized for the development of fibers consisting mainly of highly oriented stereocomplex crystals. Furuhashi et al. [11] reported that fibers drawn at various temperatures exhibit either amorphous, highly oriented homocrystals or a mixture of homocrystals and stereocomplex crystals with a fairly low orientation, depending on the drawing temperature. Annealing of the drawn fibers at an elevated temperature higher than the melting temperature of homocrystals increases the stereocomplex crystal content significantly. Masaki et al. [12] reported that PLLA and PDLA with high molecular weights can be melt blended and melt spun into amorphous fibers. Some of the drawn fibers exhibit very broad wide-angle X-ray diffraction (WAXD) reflections from the homocrystals, which immediately transform to stereocomplex crystals in the annealing process at elevated temperatures without relaxing the molecular orientation of PLLA and PDLA. Annealing under tension seems to extend the tie molecules between the lamella of the stereocomplex crystal, and the structural change significantly reflects the mechanical property. Xiuqin Zhang et al. [13] also show that highly oriented stereocomplex crystals can be formed in PLLA/PDLA blend fibers drawn at 60 °C and annealed at 200 °C. However, at a drawn temperature of 80 °C, only lower oriented stereocomplex crystals can be formed. For PLLA/PDLA blend fibers drawn twice at 60 °C, the crystallinity of stereocomplex crystals increases with annealing temperature in the range of 200 to 215 °C, while the degree of orientation decreases slightly. However, Stoclet [14] found that the stereocomplex crystal forms cannot be induced to form a PLLA/PDLA blended film through stretching; the stereocomplex form is induced at elevated crystallization temperatures, namely, above the melting temperature of the homocrystal. Lee et al. [15] developed solution-spun fibers with high crystallization during the coagulation process in solution spinning, and only homocrystals of PLLA and PDLA were detected. Drawing of the fiber at temperatures between 80 and 160 °C did not promote stereocomplexation. The drawn fiber exhibited two homocrystal phases: One easily transformed to a stereocomplex crystal upon annealing at an elevated temperature, and the other either remained as a homocrystal or froze into the amorphous phase.

Modulated differential scanning calorimetry (MDSC) has been proven to be a useful technique for understanding various thermal events and for analyzing them more precisely where standard DSC fails to separate some overlapping processes [16]. Solarski et al. [17] determined more precisely the influence of hot drawing on glass transition, relaxation, crystallization, and melting by analyzing the reversing and non-reversing MDSC curves. Gracia-Fernández et al. [18] also measured the heat capacity change during cold crystallization and correctly interpreted events at the reported double melting peak of PLLA by using experimental conditions suitable for MDSC. The formation of homocrystals and stereocomplex crystals of the oriented PLLA/PDLA fibers is interesting, but is not completely understood. In this work, a series of PLLA/PDLA blended chips were prepared for fiber formation in the melt spinning process. The as-spun PLLA/PDLA blended fibers were subjected to a hot drawing process. MDSC, hot stage polarized microscopy (HSPM), and real-time WAXD were used to investigate the homocrystallization and stereocomplex crystallization of the as-spun and hot-drawn PLLA/PDLA blended fibers during the heating process. The movement of the fiber molecular chains and the molecular chain rearrangement and transformation of the molten PLLA and PDLA to form stereocomplex crystals were discussed in this work.

## 2. Materials and Methods

PLLA (Synterra PLLA 1510) and PDLA (Synterra PDLA 1010) were supplied in chip form by Synbra Technology BV (Etten-Leur, The Netherlands). The initial thermal degradation temperatures of PLLA and PDLA were 305 °C and 307 °C, respectively, as measured by thermogravimetric analysis (TGA2025, Du-Pont, Delaware, USA). A series of PLLA/PDLA blends were prepared through melt-blending various PLLA:PDLA chip weight ratios in a mixer at 210 °C with a rotation speed of 100 rpm and a throughput speed of 8 k/h. The feed ratios of PLLA:PDLA weights were 90:10, 80:20, 70:30, 60:40, 50:50, 40:60, 30:70, 20:80, and 10:90. Using a laboratory-scale melt spinning system, the as-spun LD_A_ fiber was obtained at a spinning temperature of 250 °C with a take-up velocity of 2 km/min and a 36-hole spinneret. The hot-drawn LD_H_ fiber was prepared in a stretching machine of a set of drawing rolls and a heating chamber at a draw ratio of 1.6 to 2.1. The drawing temperature was 105 °C. The compositions of the LD blends used in this study are listed in Table 1.

The thermal properties of the samples were investigated using a differential scanning calorimeter (DSC Q100, Du-Pont, Delaware, USA) with a modulation and a thermal analysis system (TA2000, Delaware, USA). In and Pb were used for temperature calibration. Sapphire was used for heat capacity calibration. N_2_ was used as the purge gas at a flow rate of 50 cm^3^/min. The sample weight was 4–6 mg.

The morphology and structure evolution of the fibers during heating were observed using a polarizing microscope (BX51, Olympus, Tokyo, Japan) with a heater (THMS 600, Linkam, Surrey, UK), an electric microscope controller (TMS91, Linkam, Surrey, UK), and a digital camera (SPOT Idea ID2820, Michigan, USA). The flow rate of N_2_ was 120 cm^3^/min. The heating rate was 10 °C/min. The birefringence of the fibers was measured using a polarizing microscope (BX51, Olympus, Tokyo, Japan) with a Berek compensator (U-CTB: 0–11,000 nm, Olympus, Tokyo, Japan) and a monochromatic light absorption filter (550-IF: 546.1 nm, Olympus, Tokyo, Japan).

Endstation BL17A1 (wavelength, λ = 1.333 Å) of the Nation Synchrotron Radiation Research Center, Hsinchu, Taiwan, was used to observe the crystalline structures of LD_C_ chips, as-spun LD_A_ fibers, and hot-drawn LD_H_ fibers. The diffraction patterns were recorded using a Mar345 imaging plate area detector (Fuji Bas 2500 IP, Bicron). The reflection profile was deconvoluted using Jade 6 curve-fitting software (Livermore, CA, USA) with the peak search method, and a full width at half maximum value of 3 was applied to separate the amorphous phase and crystal reflections.

## 3. Results and Discussion

**Figure 1:**
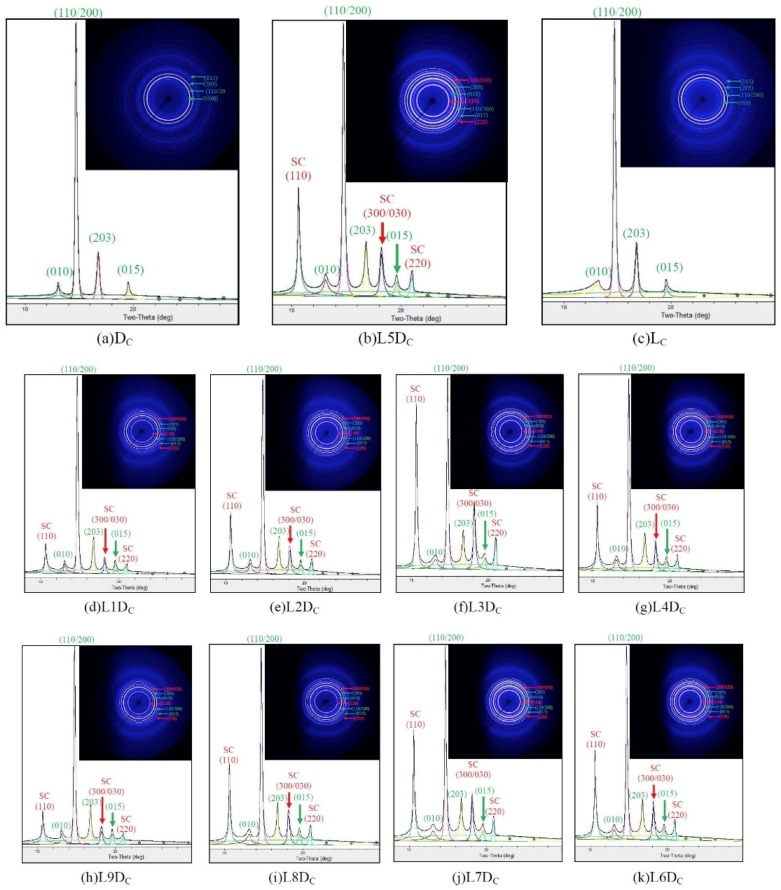
1D wide-angle X-ray diffraction (WAXD) profiles and 2D WAXD patterns for L_C_, D_C_, and LD_C_ chips. (010), (110/200), (203), and (015) in green are related to homocrystal reflections; (110), (300/030), and (220) in red are related to stereocomplex crystal reflections.

For WAXD crystalline structure analysis, the L_C_, D_C_, and LD_C_ chips were first melted at 290 °C for 3 min, then quickly cooled to 140 °C, and then subjected to isothermal crystallization for 120 min. A heater (THMS 600, Linkam) equipped with an electric microscope controller (TMS91, Linkam) was used for sample preparation. A series of 1D WAXD profiles with deconvoluted reflections and 2D WAXD patterns for the L_C_, D_C_, and LD_C_ chips are provided in Figure 1. L_C_ and D_C_ exhibited four 2θ diffraction peaks in the 1D profiles and four diffraction rings in the 2D patterns, which related to (010), (110/200), (203), and (015) planes of the homocrystals. For the LD_C_ chips, seven 2θ diffraction peaks were seen, including (110), (010), (110/200), (203), (300/030), (015), and (220) planes from the center of the 2D profile outwards. Here, (110), (300/030), and (220) planes belonged to a stereocomplex crystal.

**Figure 2:**
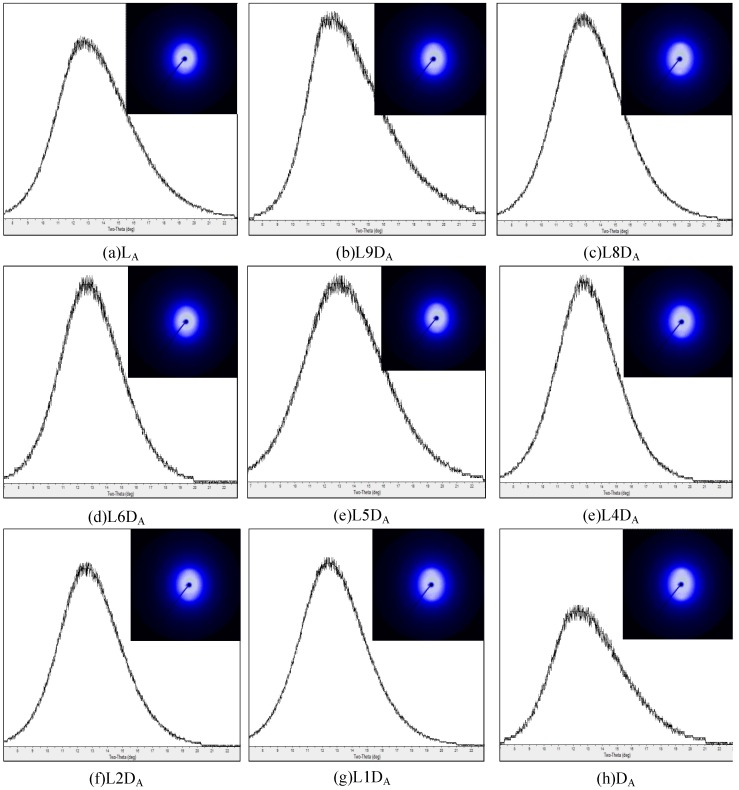
1D WAXD profiles and 2D WAXD patterns for as-spun fibers.

**Figure 3:**
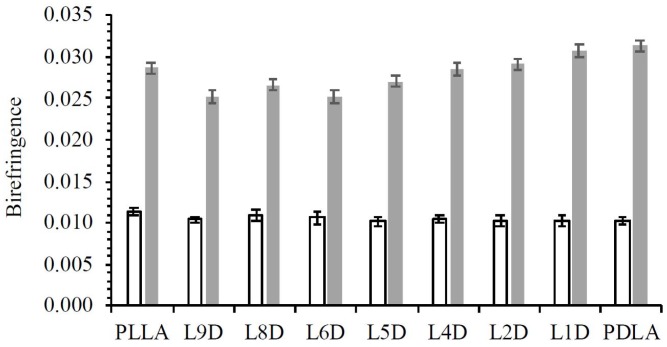
Birefringence of as-spun (white) and hot-drawn (gray) fibers.

**Figure 4:**
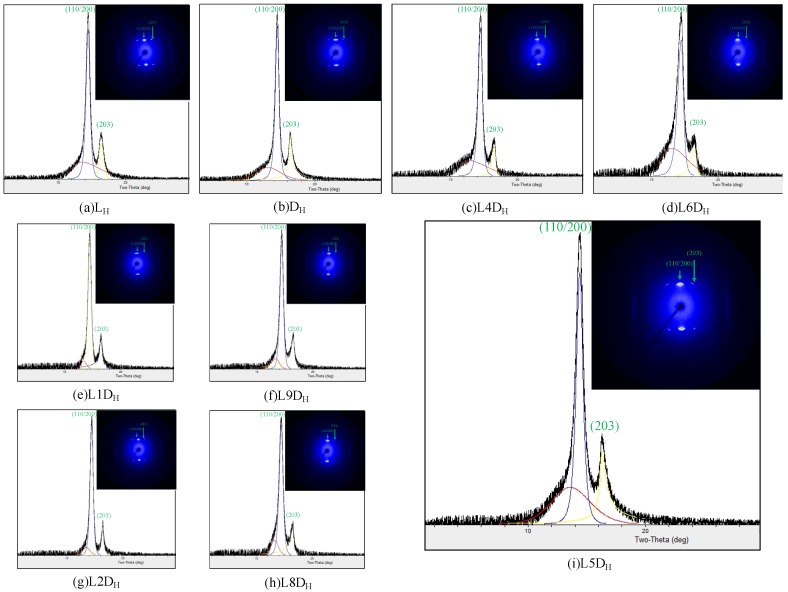
1D WAXD profiles and 2D WAXD patterns for hot-drawn fibers. (110/200) and (203) are homocrystal reflections.

1D WAXD profiles and 2D WAXD patterns for the as-spun fibers are depicted in Figure 2. No sharp diffraction peak occurred in the 1D WAXD profiles of any as-spun fibers, only an amorphous peak. The 2D WAXD patterns of the non-crystalline as-spun fibers appeared to exhibit a certain degree of microstructural alignment along the fiber direction. The birefringence of the as-spun fibers is depicted in Figure 3. This indicates that the molecular chains were slightly oriented during the fiber spinning process. The birefringence values of L_A_ and D_A_ were not significantly different from those of LD_A_. This means that whether molecular chains were packed side by side or in the solely L or D form did not affect the orientation of molecular chains during spinning at a temperature of 250 °C at a take-up velocity of 2 km/min. The molecular chain orientation of the as-spun fiber was further improved during the hot drawing process (Figure 3). The birefringence values of L_H_ and D_H_ were slightly higher than those of LD_H_. This means that, in the hot drawing process at a temperature of 105 °C and a draw ratio of 1.6 to 2.1, the molecular chain of the L_H_ or D_H_ fiber consisting solely of L or D form was slightly easier to orient than the molecular chain of the LD_H_ fiber packed side by side. Two diffraction arcs (110/200) and (203) related to homocrystals were observed on all of the hot-drawn fibers (Figure 4). No stereocomplex crystal was observed in all of the hot-drawn fibers. This indicates that molecular chain orientation is improved after the hot-drawing process, which induces crystal formation. Here, the hot-drawing temperature was set at 105 °C, which was advantageous for homocrystallization.

**Figure 5:**
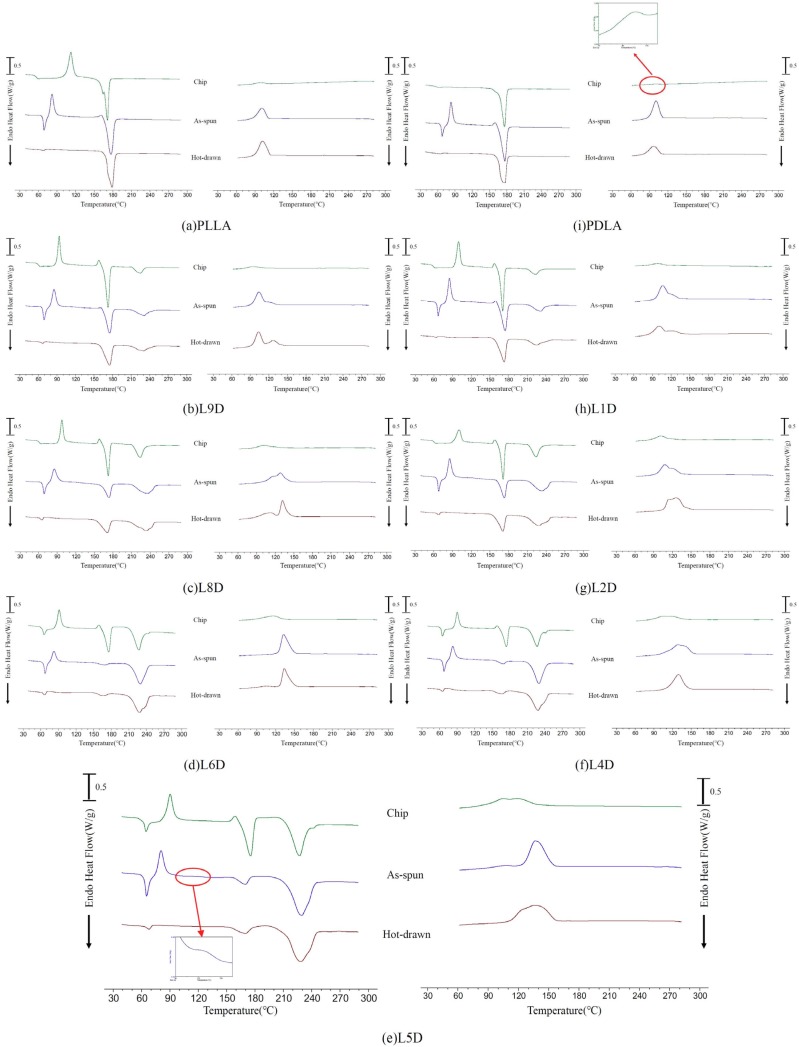
Standard differential scanning calorimeter (DSC) thermal analyses of chips, as-spun fibers, and hot-drawn fibers. Samples were heated to 290 °C for 3 min at 10 °C/min and then cooled to 30 °C at 10 °C/min.

**Figure 6:**
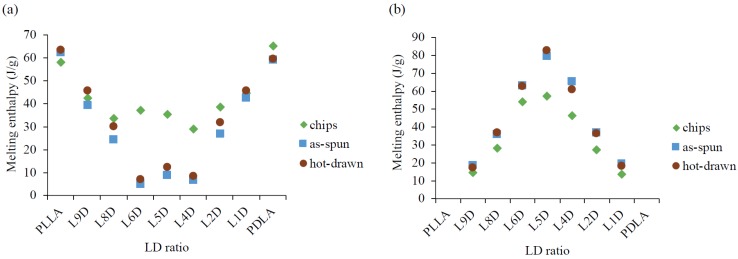
Melting enthalpy of the homocrystal and the stereocomplex crystal of the chips, as-spun fibers, and hot-drawn fibers. (**a**) Δ*H*_m_ of a homocrystal and (**b**) Δ*H*_m_ of a stereocomplex crystal.

The standard DSC thermal analyses of the chips, as-spun fibers, and hot-drawn fibers are presented in Figure 5. The heating and cooling rates were set at 10 °C/min. Compared with LD_C_, an increase in T_g_ for the corresponding as-spun fiber in the heating curve was observed. Glass transition of the as-spun LD_A_ fibers was immediately followed by an endothermic peak, which was related to the relaxation of molecular chains. The relaxation enthalpy of the as-spun fiber was significantly higher than that of the corresponding chip, which means that the mobility of the oriented molecular chains after spinning was restricted. The cold crystallization peaks of all of the as-spun fibers occurred just after the relaxation phenomenon in the heating curve. Compared with LD_C_, the cold crystallization peak of the corresponding LD_A_ occurred earlier in the heating curve. This was because the molecular chains were oriented during spinning and then induced to crystallize during heating at lower temperatures. As the heating curve of the LD_A_ fiber was enlarged, a small second cold crystalline shoulder was observed; the L5D_A_ fiber is exemplified in Figure 5e. Two melting peaks associated with homocrystals and stereocomplex crystals were observed in all LD_A_ fibers. The homocrystal melting enthalpy of the L_A_ and D_A_ fibers was almost the same as that of the corresponding L_C_ and D_C_ chips, but the homocrystal melting enthalpy of the LD_A_ fiber was lower than that of the corresponding LD_C_ chip. The homocrystal melting enthalpy difference between the LD_C_ chip and the corresponding LD_A_ fiber increased as the stereocomplex crystal melting enthalpy of the LD_A_ fiber increased (Figure 6). After hot drawing at 105 °C, the relaxation enthalpy of the LD_H_ fiber was considerably lower than that of the corresponding LD_A_ fiber. This means that the mobility of the fiber molecular chains after hot drawing was further restricted by the formation of more oriented molecular chains and homocrystals. The molecular chains were stabilized in a state that did not evolve much after the glass transition temperature. The melting enthalpy of the homocrystal and stereocomplex crystal of the LD_H_ fiber was almost the same as that of the corresponding LD_A_ fiber.

A single exothermic peak of L_C_ and D_C_ in the cooling curve was approximately 100 °C, which was contributed by the homocrystallization of PLLA and PDLA (Figure 5a,i). A broad exothermic peak was observed in all LD_C_ chips during cooling, possibly mixing the formation of homocrystals and stereocomplex crystals (Figure 5b–h) [5,19,20,21]. The crystallization enthalpy of the LD_C_ chips during cooling was significantly higher than that of the L_C_ and D_C_. This is because the stereocomplex crystal of the LD_C_ chips that formed beforehand acted as nucleating agents, and the crystallization temperature periods of the homocrystals and stereocomplex crystals overlapped [5]. When the as-spun and hot-draw fibers were heated to 290 °C, which was higher than the equilibrant melting temperature of stereocomplex crystals at 279 °C [22], and then cooled, the crystallization enthalpy of all the as-spun and hot-drawn fibers during cooling was significantly higher than that of the corresponding chip. This indicates that the orientation of the existing molecular chains on the as-spun and hot-draw fibers after heating at 290 °C for 3 min was not completely disordered, which was advantageous for crystallization during cooling.

**Figure 7:**
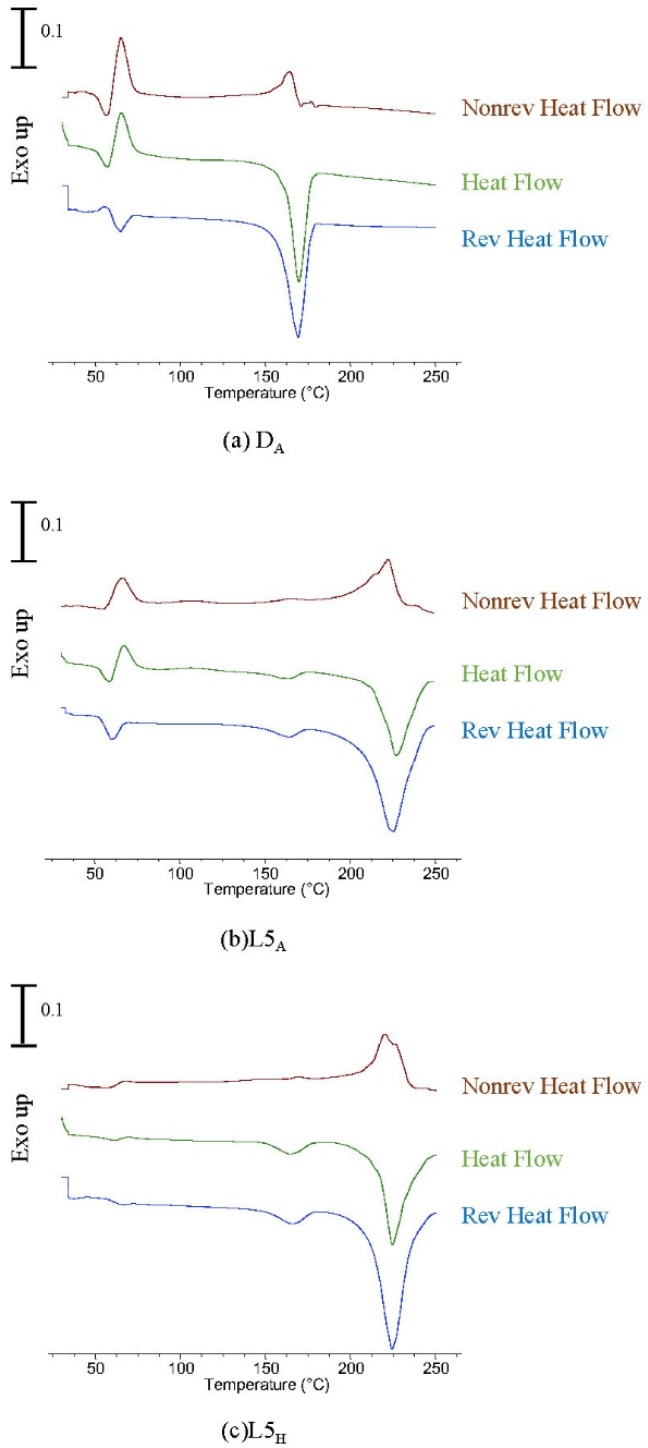
Modulated DSC (MDSC) curves obtained at a heating rate of 2 °C/min, a period of 200 s, and an amplitude of 1 °C. (**a**) D_A_, (**b**) L5D_A_, and (**c**) L5DH.

To further investigate the crystallization and melting of the homocrystals and stereocomplex crystals during heating, the MDSC measurements were carried out at an underlying heating rate of 2 °C/min, an amplitude of 1 °C, and a period of 200 s. D_A_, L5D_A_, and L5D_H_ were used as examples. These parameters allow observation of the glass transition and melting in the reversing curve and of relaxation and crystallization in the non-reversing curve (Figure 7). The exothermic peak of D_A_ at 65 °C immediately after the relaxation phenomenon was observed clearly in the non-reversing curve (Figure 7a). The exotherm was attributed to the cold crystallization of homocrystals. It was related to *α*′ crystal formation [23]. An additional exotherm was observed on the non-reversing curve between approximately 105 °C and 165 °C. On the reversing curve, the melting temperature of the homocrystal was in the range of 130 °C to 180 °C. Due to the *α*′-to- *α* transition in the range of 100 °C to 120 °C, the recrystallization and melting behaviors of the D_A_ homocrystal overlapped in the range of 130 °C to 165 °C. In the L5D_A_ non-reversing curve, the first exotherm was attributed to the cold crystallization of homocrystals just after the relaxation peak; the second exotherm at approximately 90 °C to 125 °C was related to the cold crystallization of stereocomplex crystals (Figure 7b). A significant exotherm began at approximately 130 °C to 220 °C, which combined the recrystallization of the homocrystal and the stereocomplex crystal. On the reversing curve, the melting began at approximately 130 °C for the homocrystal and at approximately 180 °C for the stereocomplex crystal. The melting peaks of the homocrystal and stereocomplex crystal were 165 °C and 225 °C, respectively. The cold crystallization enthalpy of the stereocomplex crystal at approximately 90 °C to 125 °C was significantly lower than the melting enthalpy of the stereocomplex crystal. This means that the stereocomplex crystal was formed mainly during recrystallization. The molecular chains of the melted PLLA and PDLA homocrystals were rearranged and transited to form stereocomplex crystals. The recrystallization and melting behaviors of the stereocomplex crystal overlapped in the range of 170 °C to 220 °C.

After hot drawing at 105 °C, the relaxation enthalpy of the L5D_H_ fiber was much lower than that of the L5D_A_ fiber (Figure 7c). Cold crystallization and recrystallization of the homocrystal and the stereocomplex crystal of L5D_H_ were still observed on the non-reversing curve, and the enthalpy of the cold crystallization of the L5D_H_ homocrystal and stereocomplex crystal was significantly smaller than that of the L5D_A_ fiber (Figure 7b,c). However, the melting enthalpy of the homocrystal and stereocomplex crystal of the L5D_H_ fiber was almost the same as that of the corresponding L5D_A_ fiber (Figure 7).

According to standard DSC thermal analyses, the homocrystal melting enthalpy of L_A_ and D_A_ was significantly higher than that of LD_A_ (Figure 5). The reversing curves of MDSC confirmed that the homocrystal melting enthalpy of D_A_ was significantly higher than that of L5D_A_ (Figure 7). This is because, when the chips were compressed, heated, and melted using an extrusion screw, the molecular chains were further mixed and then fed to a spinning pump and into a spinneret to form fibers. The molecular chains were uniformly mixed using the extrusion screw, facilitating side-by-side packing of the molecular chains. This resulted in a lower melting enthalpy for the homocrystal of the LD_A_ fiber than of the corresponding LD_C_ chip and a higher melting enthalpy for the stereocomplex crystal of the LD_A_ fiber than of the corresponding LD_C_ chip (Figure 6). This indicates that when the l- or d- molecular chains were mixed sufficiently as to become packed side by side, the LD_A_ had more stereocomplex crystals than homocrystals after heating.

**Figure 8:**
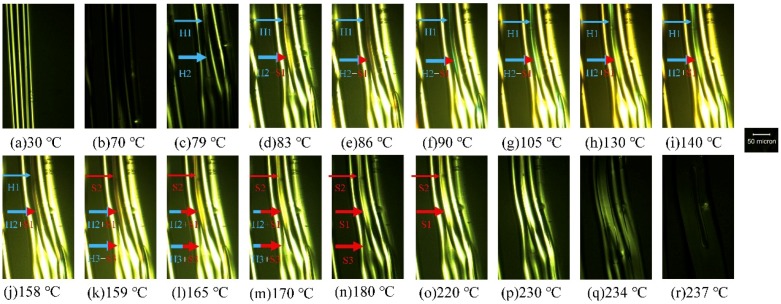
Polarized photographs of the as-spun L5D_A_ fibers at elevated temperatures, taken at the temperature indicated below the image. Markers H and S represent the homocrystal and stereocomplex crystal, respectively.

According to the WAXD study and birefringence analysis, all LD_A_ fibers exhibited an amorphous state with a slight molecular chain orientation. DSC results revealed that as-spun LD_A_ fibers’ homocrystals and stereocomplex crystals were formed during heating. The phase transition of the LD_A_ fiber from an amorphous state to a crystalline state during heating was observed using HSPM. Here, the L5D_A_ fiber was used as an example. Figure 8 depicts the temperature dependence of polarized light photographs of L5D_A_ fibers. The optical anisotropy of L5D_A_ fibers was seen in all of the fibers at room temperature, which was due to the orientation of the molecular chains. As the temperature increased to 70 °C, after the glass transition temperature of L5D_A_, the optical anisotropy of the fiber approached darkness (Figure 8b), where molecular chains of the fibers relaxed. While the fibers were continuously heated, they gradually became brighter due to the cold crystallization of homocrystal and stereocomplex crystals until 130 °C (Figure 8c–h). Figure 8d illustrates the homocrystal forms, indicated by arrow H_1_ and the homocrystal in combination with the stereocomplex crystal form indicated by arrow H_2_+S_1_. Here, the marker H and the marker S represent the homocrystal and stereocomplex crystal, respectively. When the temperature exceeded 140 °C, some parts of the fibers gradually darkened and then relighted in a temperature range of about 140 °C to 165 °C. The change in brightness can be seen as indicated by the arrows H_1_ and S_2_ in Figure 8i–l. Here, the color change from bright to dark was due to the melting of the homocrystal (see arrows H_1_ and H_2_+S_1_ in Figure 8i–j), and the color was relighted due to the recrystallization of the homocrystal and stereocomplex crystal (see arrows S_2_ and H_2_+S_1_ in Figure 8k). When the temperature exceeded 159 °C, the brightness was slightly reduced to 180 °C (see arrow H_3_+S_3_ Figure 8k–n); however, the brightness of the portion originally marked as H_1_ was relighted (see arrow S_2_ Figure 8k–m). Here, the color change should be related to the melting of the homocrystal, and the brightness of the portion originally marked as H_1_ is relighted by the molecular chain rearrangement and recrystallization to form the stereocomplex crystal. The area marked H_1_ thus became as seen in the area indicated by S_2_. Subsequently, the brightness of some parts gradually increased again until 220 °C (see arrows S_1_ and S_2_ in Figure 8n–o). This relates to the recrystallization of the stereocomplex. When the temperature increased to 237 °C (Figure 8r), all stereocomplex crystals melted almost completely. The polarized photographs of the L5D_A_ fibers corresponded closely with the exotherm and endotherm behaviors of the L5D_A_ heating curve. The change in brightness and color seen in the polarized photographs of L5D_A_ fibers during heating clearly shows the molecular chain movement of the fibers, including molecular chain orientation and relaxation; as well as crystallization, melting, and recrystallization of the homocrystal, and rearrangement, recrystallization, and melting of the stereocomplex crystal.

**Figure 9:**
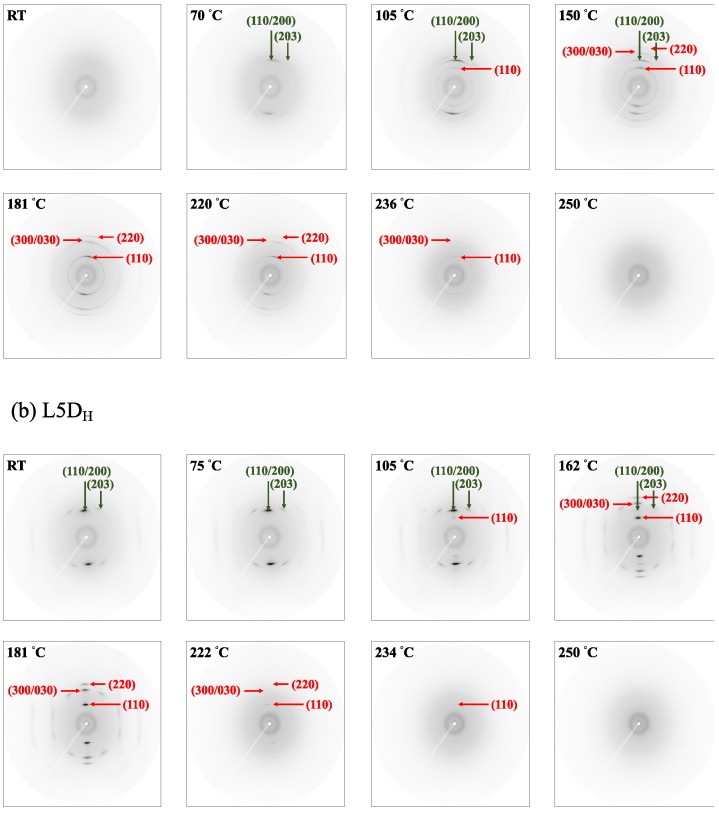

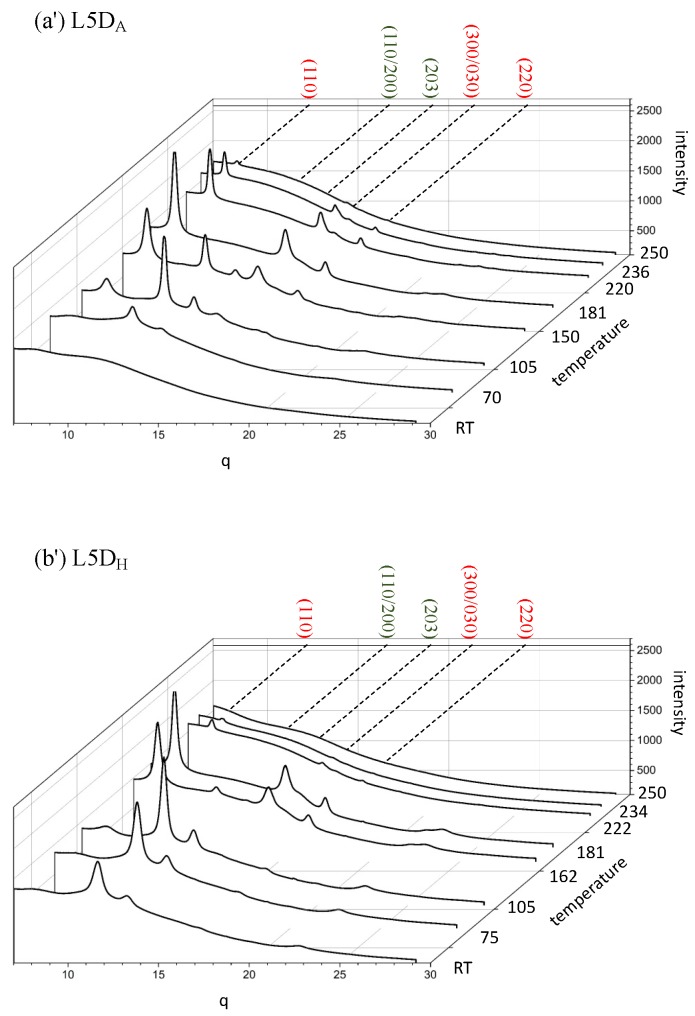
2D WAXD patterns and corresponding intensity curves of L5D_A_ and L5D_H_ fibers, captured at the temperature indicated in the image.

The homocrystallization and stereocomplex crystallization of the as-spun and hot-drawn fibers during heating was further investigated using a real-time WAXD measurement. The as-spun L5D_A_ fiber and the hot-drawn L5D_H_ fiber were respectively wrapped with Kepton and heated from room temperature to near complete melting at a rate of 10 °C/min. When the temperature was increased to the set temperature, the sample was equilibrated for 1 min and subjected for 3 min to X-ray recording. Figure 9a shows the 2D WAXD patterns of the L5D_A_ fiber obtained at the set temperature, and the corresponding intensity curve is depicted in Figure 9a’. The WAXD pattern illustrates that the L5D_A_ fiber was in the amorphous state at room temperature. When the fiber was heated to 70 °C, two weak (110/200) and (203) reflections related to the homocrystal appear in the L5D_A_ fiber. At temperatures of 70 °C to 105 °C, the diffraction intensities of the reflections (110/200) and (203) increased, and a new weak (110) reflection associated with the stereocomplex crystal appeared. This indicates that the temperature was higher than the T_g_ of L5D_A_, the molecular chain of the fiber relaxed and then crystallized, and the homocrystal and stereocomplex crystal were sequentially formed. As the temperature was further increased to 150 °C, (300/030) and (220) reflections associated with the stereocomplex crystals appeared; however, the diffraction intensities of the (110/200) and (203) peaks decreased. The melting of homocrystals was combined with the formation of stereocomplex crystals. When the temperature reached 181 °C, the homocrystal completely melted. The diffraction intensities of (110), (300/030), and (220) peaks increased to 181 °C and then gradually decreased. All reflections almost disappeared at 250 °C. When the temperature exceeded T_g_, the homocrystal and stereocomplex crystal formed sequentially; and during the heating at approximately 105 °C to 180 °C, stereocomplex crystal formation occurred simultaneously with the melting of the homocrystal.

The 2D WAXD patterns of the L5D_H_ fiber obtained at the set temperatures and the corresponding intensity curves are depicted in Figure 9b,b’. The L5D_H_ fiber had two (110/200) and (203) reflection arcs associated with the homocrystal. When the fiber was heated to 75 °C, the diffraction intensities of (110/200) and (203) peaks increased. At temperatures of 75 °C to 105 °C, the diffraction intensities of (110/200) and (203) peaks increased, and a new weak (110) reflection associated with the stereocomplex crystal appeared. When the temperature was further increased to 162 °C, (300/030) and (220) reflections associated with the stereocomplex crystals appeared; however, the diffraction intensities of (110/200) and (203) peaks were significantly lowered. The (110/200) and (203) reflections related to the homocrystal continued to exist from 162 °C to 181 °C. The diffraction intensities of (110), (300/030), and (220) peaks increased to 181 °C, then gradually decreased and all reflections disappeared completely at 250 °C. The results demonstrate that stereocomplex crystallization of L5D_H_ was almost the same as that of L5D_A_. All L5D_H_ fibers exhibited arc reflections, and all L5D_A_ fibers exhibited ring reflections. This indicates that the molecular chain orientation of the L5D_A_ fiber was improved after the hot-drawing process.

## 4. Conclusions

In this research, a series of the LD chips, as-spun fibers, and hot-drawn fibers were prepared for studying homocrystallization and stereocomplex crystallization during heating. WAXD analysis showed that the as-spun LD_A_ fibers obtained at a take-up velocity of 2 km/min did not crystallize during spinning at 250 °C. All of the as-spun LD_A_ fibers were in an amorphous state with a slight molecular chain orientation along the fiber axis. Compared to the chips, the oriented molecular chains of the corresponding as-spun fiber are induced to form homocrystal and stereocomplex crystals earlier in the subsequent heating process. The cold crystallization related to the homocrystal occurred just after the molecular chain relaxation of the as-spun fiber, and the recrystallization and melting behaviors of the homocrystal of the as-spun fiber then overlapped in the range of 130 °C to 180 °C. As the molecular chains were further mixed by the extrusion screw during the spinning process, side-by-side packing of the molecular chains was promoted. This resulted in the melting enthalpy of the homocrystal of the as-spun LD_A_ fiber being lower than that of the corresponding LD_C_ chip, and the melting enthalpy of the stereocomplex crystal of the as-spun LD_A_ fiber was higher than that of the corresponding LD_C_ chip. The cold crystallization of the stereocomplex occurred just after the cold crystallization of the homocrystal of the as-spun fiber. The MDSC results indicated that the stereocomplex crystal was formed mainly during recrystallization, which means that the molecular chains of the molten PLLA and PDLA were rearranged and transited to form stereocomplex crystals; the recrystallization and melting behaviors of the stereocomplex crystal overlapped in the range of 170 °C to 220 °C.

HSPM observations, MDSC analysis, and real-time WAXD measurements indicated that the molecular chain of the fiber relaxed when the temperature exceeded *T*_g_. The homocrystal and stereocomplex crystal were sequentially formed just after the relaxation phenomenon and subsequent melting and recrystallization of the homocrystal. The molecular chains of the molten PLLA and PDLA were rearranged and transited to form the stereocomplex crystal, and then melting and recrystallization of the stereocomplex crystal occurred. Compared with standard DSC, MDSC more accurately separated the homocrystallization and stereocomplex crystallization behaviors, and HSPM photographs closely matched to MDSC results.

## Figures and Tables

**Table 1:** Composition of samples.

Sample Type			PLLA (wt%)	PDLA (wt%)
Chip	As-Spun Fiber	Hot-Drawn Fiber		
L_C_	L_A_	L_H_	100	0
L9D_C_	L9D_A_	L9D_H_	90	10
L8D_C_	L8D_A_	L8D_H_	80	20
L7D_C_	-	-	70	30
L6D_C_	L6D_A_	L6D_H_	60	40
L5D_C_	L5D_A_	L5D_H_	50	50
L4D_C_	L4D_A_	L4D_H_	40	60
L3D_C_	-	-	30	70
L2D_C_	L2D_A_	L2D_H_	20	80
L1D_C_	L1D_A_	L1D_H_	10	90
D_C_	D_A_	D_H_	0	100

L7DA and L3DA cannot be wound continuously.
